# Validity of Computer-interpreted “Normal” and “Otherwise Normal” ECG in Emergency Department Triage Patients

**DOI:** 10.5811/westjem.58464

**Published:** 2023-12-06

**Authors:** Ashley Deutsch, Kye Poroksy, Lauren Westafer, Paul Visintainer, Timothy Mader

**Affiliations:** * University of Massachusetts Chan Medical School, Baystate Medical Center, Springfield, Massachusetts

## Abstract

**Introduction:**

Chest pain is the second most common chief complaint for patients undergoing evaluation in emergency departments (ED) in the United States. The American Heart Association recommends immediate physician interpretation of all electrocardiograms (ECG) performed for adults with chest pain within 10 minutes to evaluate for the finding of ST-elevation myocardial infarction (STEMI). The ECG machines provide computerized interpretation of each ECG, potentially obviating the need for immediate physician analysis; however, the reliability of computer-interpreted findings of “normal” or “otherwise normal” ECG to rule out STEMI requiring immediate intervention in the ED is unknown.

**Methods:**

We performed a prospective cohort analysis of 2,275 ECGs performed in triage in the adult ED of a single academic medical center, comparing the computerized interpretations of “normal” and “otherwise normal” ECGs to those of attending cardiologists. ECGs were obtained with a GE MAC 5500 machine and interpreted using Marquette 12SL.

**Results:**

In our study population, a triage ECG with a computerized interpretation of “normal” or “otherwise normal” ECG had a negative predictive value of 100% for STEMI (one-sided, lower 97.5% confidence interval 99.6%). None of the studied patients with these ECG interpretations had a final diagnosis of STEMI, acute coronary syndrome, or other diagnosis requiring emergent cardiac catheterization.

**Conclusion:**

In our study population, ECG machine interpretations of “normal” or “otherwise normal” ECG excluded findings of STEMI. The ECGs with these computerized interpretations could safely wait for physician interpretation until the time of patient evaluation without delaying an acute STEMI diagnosis.

Population Health Research Capsule What do we already know about this issue?

*The American Heart Association recommends screening ED triage electrocardiograms (ECG) within 10 minutes for evidence of ST-elevation myocardial infarction (STEMI).*

What was the research question?

*What is the reliability of an ECG machine interpretation of a “normal” or “otherwise normal” ECG in ruling out STEMI?*

What was the major finding of the study?

*The negative predictive value for STEMI of ECGs with these interpretations is 100% (one-sided, lower 97.5% confidence interval limit: 99.6%).*

How does this improve population health?

*This study further confirms that physician interpretation of triage ECGs with these computerized interpretations may be safely deferred until the time of patient evaluation.*

## INTRODUCTION

### Background

Each year there are more than nine million emergency department (ED) visits for acute nontraumatic chest pain in the United States.^1^ This is the second most common chief complaint for patients undergoing emergent evaluation.^2^ Expedited identification of life-threatening, acute ST-segment elevation myocardial infarction (STEMI), a diagnosis made solely by recognition of characteristic patterns of heart injury on electrocardiogram (ECG), is critical to timely intervention and optimal patient outcomes. The current American Heart Association (AHA) recommendation is for all ED chest pain ECGs to be obtained within 10 minutes of patient arrival and immediately screened for STEMI by a clinician.^3^ Computerized software algorithms can analyze and print a preliminary ECG interpretation in real time; however, the interpretation algorithms are proprietary and manufacturer-specific.^4^ ^–^ ^6^ The degree of variability in diagnostic accuracy among computer programs was significantly greater than that among cardiologists.^5^ ^–^ ^7^

### Importance

Approximately 60% of triage ECGs at our institution are interpreted as “borderline” or “abnormal” and necessitate immediate clinician screening for acute coronary syndrome (ACS) and possible STEMI. The remainder are interpreted as “normal” or “otherwise normal” ECG (eg, sinus bradycardia-otherwise normal) by the computer. There are limited studies investigating whether these latter readings are reliable in ruling out STEMI.^8^ ^–^ ^10^ Recent evidence suggests that computerized interpretation of normal sinus rhythm/normal ECG—the so called “normal/normal”—has a negative predictive value (NPV) of 99% (confidence interval [CI] 97–99%) with no reported cases of missed ACS or STEMI, which may obviate the need for immediate clinician verification.^9^ ^,^ ^10^ The study provided some insights into the reliability of these interpretations but included small numbers of ECGs and did not evaluate ECGs read as “otherwise normal.”

We reasoned that while immediate physician interpretation of ECGs in patients with chest pain is recommended by the AHA to screen for ECGs that meet STEMI criteria, it may not be necessary in some triage ECGs. To understand the impact of delaying immediate interpretation to the time of patient encounter, it is important to understand whether this delay would potentially delay diagnosis of this time-sensitive finding.

### Goals of this Investigation

We performed a prospective cohort study of all adult triage patients in our ED who received an ECG during the study period to compare the computerized ECG interpretation of “normal” or “otherwise normal” ECG to that of the attending cardiologist. Our aim was to determine the NPV of these computerized interpretations for STEMI and ECG signs of acute ischemia.

## MATERIALS AND METHODS

### Study Design and Setting

This was a prospective cohort study of triage ECGs performed by patient care technicians or triage nurses according to the standard triage protocol in the adult ED of a large academic hospital. This ED is one of the busiest in the Northeast US, serving a population base of over one million people and caring for more than 130,000 patients annually, of whom approximately 8,000 have a chief complaint of acute chest pain. Our medical center is the regional tertiary-care facility for interventional cardiology, and it is the second busiest interventional cardiology lab in the state. This study was approved by the institutional review board. We have adhered to the Strengthening of Reporting of Observational Studies in Epidemiology (STROBE) statement.

### Selection of Participants

**Figure 1.**
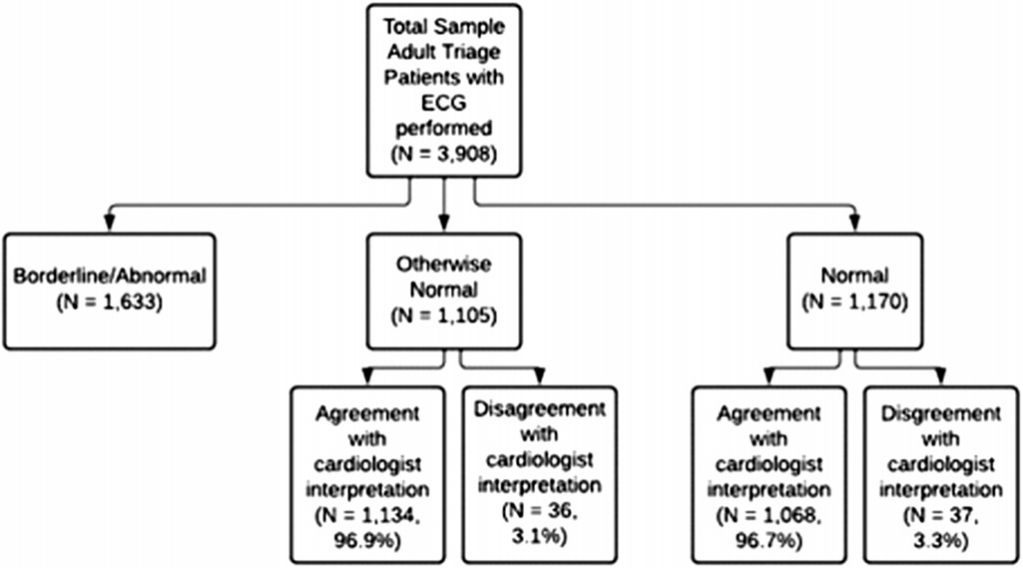
Results of a comparison of computer-read vs cardiologist interpretation of electrocardiograms performed at triage.

We included all patients ≥18 years old who had a triage ECG performed by patient care technicians (PCT) or triage nurses according to a standard triage protocol in the adult ED (≥18 years of age). The nurse triage ECG protocol required obtaining an ECG on patients with a chief complaint of chest pain, chest pressure, chest tightness, weakness, unusual fatigue, palpitations, syncope, dyspnea, or any atypical symptoms consistent with ACS such as nausea and vomiting or pain in the jaw, upper back, or upper abdomen. The ECGs were collected at all hours of the day seven days per week from June 2018–October 2021, with recruitment paused for approximately 18 months due to the COVID-19 pandemic (Figure 1). There were no changes in ECG protocols or cardiac catheterization lab protocols during that time.

### Interventions

Triage ECGs were obtained per protocol and immediately presented to an attending emergency physician for review. Upon return to triage, PCTs printed a copy of the ECG and placed it in a collection box for research staff. The screened ECGs were then placed in the patients’ charts for the treating physicians to review at the time of the patient evaluation. The ECGs performed according to triage protocol during the designated study period were prospectively collected by research associates.

The ECGs were obtained with a GE MAC 5500 (GE Healthcare, Waukesha, WI) and interpreted using Marquette 12SL (GE Healthcare). The ECGs were uploaded to a secure hospital server. Board-certified cardiologists blinded to all aspects of the study reviewed the ECGs and entered the final interpretation into the medical health records as per standard operating procedure.

### Measurements

The primary outcome of interest was the number of ECGs with a computerized interpretation of “normal” ECG or “otherwise normal” ECG that were interpreted by a cardiologist as STEMI. Secondarily, we examined the number of patients who had ECGs with these computerized interpretations and an end diagnosis of ACS or STEMI, or had a cardiac catheterization during their hospitalization for that index visit.

A sample-size calculation demonstrated the need for at least 1,000 ECGs with a computerized interpretation of “normal” ECG and 1,000 with a computerized interpretation of “otherwise normal” ECG to adequately answer our proposed question. Given that we were evaluating a process change that would alter patient triage for ECGs in the ED, we wanted a high degree of precision in our estimates. Thus, a sample size of 3,000 records would provide a 95% CI that would be no wider than ±2 percentage points for estimates of predictive values.

All patients with a triage ECG reported as “normal” or “otherwise normal” by computer interpretation had a chart review performed by ED research associates experienced in chart review to extract patient demographics, ascertain the triage ECG indication, determine the cardiologist’s final interpretation, and document the patient’s ED disposition and final discharge diagnosis with specific attention to the presence or absence of ACS or STEMI. The data abstraction form was piloted by a research coordinator and research assistant prior to implementation. Research associates were blinded to the study hypothesis. Study data were collected and managed using REDCap electronic data capture tools hosted at our institution^11^ ^,^ ^12^ REDCap (Research Electronic Data Capture) is a secure, web-based software platform designed to support data capture for research studies, providing 1) an intuitive interface for validated data capture; 2) audit trails for tracking data manipulation and export procedures; 3) automated export procedures for seamless data downloads to common statistical packages; and 4) procedures for data integration and interoperability with external sources. Paper ECGs were kept in a secured room to reference as needed to verify the database.

We compared each ECG interpreted by the computer as “normal” or “otherwise normal” ECG to the cardiologist’s final interpretation. If the cardiologist interpretation was also “normal” or “otherwise normal” this was considered an accurate computer interpretation. If the cardiologist’s interpretation differed, we considered the computerized ECG interpretation to be inaccurate. Validation of the data entered by research staff was completed for 100% of ECGs with cardiologist disagreement (n = 74) and 15% (n = 341) randomized patients by the principal investigator (AD). We collected additional variables including gender, age, race/ethnicity, ED disposition, and final discharge diagnosis.

Finally, blinded board-certified emergency physicians were asked to evaluate any ECG with a final cardiologist interpretation of STEMI or a final diagnosis of ACS to evaluate whether ECGs would have been interpreted in real time by a clinician as indicating ACS and requiring emergent intervention.

### Outcomes

The primary outcome of this study was discordance of a computerized interpretation of “normal” or “otherwise normal” ECG, and a cardiologist interpretation of STEMI or “consider ischemia.” Secondary outcomes included final patient-encounter diagnosis of ACS and proportion of patients who received cardiac catheterization during hospitalization.

### Analysis

For descriptive analyses, continuous variables are represented with means and standard deviations. Categorical variables are presented with frequencies and proportions. Agreement or disagreement between computer and cardiologist ratings are presented as proportions with 95% CIs. Given that we selected only normal computer-read EKGs, the NPV is the only screening characteristic provided that was able to be estimated. To assess whether age or gender influenced disagreement in ratings, we compared records for which cardiologist and computer agreed to those where there was disagreement. For age, we used a *t*-test to compare the two groups on age and a chi-square test to compare the groups on gender.

## RESULTS

### Characteristics of the Study Subjects

A total of 2,275 patients were included in the study. The median age of the study population was 47 years (interquartile range [IQR] 27; IQR interval 33–60). Within the cohort, 1,262 were women (55.5%) and 73.4% were White (Table 1). The indication for ECG was chest pain in 58% of patients, followed by cardiac arrhythmia (19%). Of patients with ECG machine-interpretations of “normal” or “otherwise normal,” 98.6% were discharged from the ED. None of the patients included in the analysis had a STEMI or final diagnosis of ACS. There was no difference in mean age between the cases where there was agreement (n = 2,201) vs no agreement (n = 74). Mean age for agreement was 47.3 (±17.0) vs disagreement 50.7 (±17.9), *P* = 0.12. Similarly, no difference in agreement emerged for gender. The agreement was 96.9% (1,223/1,262) for females and 96.5% (978/1,013) for males (*P* = 0.64).

**Table 1.** Patient characteristics.

Variables (N = 2,275)	Summary
Patient Age in years Median (IQR)	47.0 (27.0)
Gender, n (%)	
Female	1,262 (55.5)
Male	1,013 (44.5)
Computer Read, n (%)	
Normal/normal	1,170 (51.4)
Otherwise/normal	1,105 (48.6)
Patient race, n (%)	
American Indian/Alaska Native	6 (0.3)
Asian	33 (1.5)
Black/African American	334 (14.7)
Hispanic/Latino	92 (4.0)
Native Hawaiian/Pacific Island	11 (0.5)
White	1669 (73.4)
Unknown/refused	130 (5.7)
ECG indication when algorithm disagrees with cardiologist (n = 74), n (%)	
Suspected acute MI, STEMI	1 (1.4)
Non-traumatic chest pain	40 (54.1)
Dyspnea	3 (4.1)
Cardiac arrhythmia	9 (12.2)
Electrolyte imbalance	1 (1.4)
Syncope	6 (8.1)
Other	8 (0.8)
Indication not provided	6 (8.1)
Hospital admission, n (%)	
No	2243 (98.6)
Yes	32 (1.4)
LWBS, n (%)	
No	2266 (99.6)
Yes	9 (0.4)
Discharge diagnosis c/w ACS, n (%)	
No	65 (89.0)
NA (LWBS/AMA/etc.)	8 (11.0)
Cardiology agree? n (%)	
Disagree	74 (3.3)
Agree	2201 (96.7)
Cardiologists reading (ECG paper read) to (cardiologists read), n (%)	
Normal/normal to otherwise normal	10 (14.1)
Normal/normal to borderline or abnormal	26 (36.6)
Otherwise normal to borderline or abnormal	35 (49.3)

*ECG*, electrocardiogram; *LWBS*, left without being seen; *AMA*, against medical advice; *STEMI*, ST-elevation myocardial infarction; *ACS*, acute coronary syndrome.

### Main Results

Cardiologists agreed with the machine-interpretation of “normal” or “otherwise normal” ECG in 96.7% (n = 2,201) of cases. Of the 3.3% (n = 74) of ECGs where cardiologists did not agree with the machine interpretation, none were interpreted by the cardiologist as STEMI. The NPV for STEMI of ECGs with these interpretations is 100% (one-sided, lower 97.5% CI limit: 99.6%). In 35 (49.3%) of the ECGs in which the cardiologists disagreed with the machine-interpretation, these ECGs were read by the machine as “otherwise normal” but the cardiologist interpreted “borderline” or “abnormal.” Ultimately none of the 2,275 patients with machine-interpreted ECGs included in the study had a discharge diagnosis of STEMI or ACS. Only 1.4% required hospital admission for any indication. Because no ECGs with these initial machine interpretations had a final interpretation of STEMI or diagnosis of ACS, further review by blinded board-certified emergency physicians was not required.

## DISCUSSION

This study found that in our triage patient population, a computerized ECG Marquette 12SL interpretation of “normal” or “otherwise normal” ECG reliably rules out a finding of STEMI. Patients who had triage ECGs with these computerized interpretations did not have a discharge diagnosis of ACS and did not require emergent catheterization. Very few patients with these ECG interpretations were admitted to the hospital. In our study population, a computerized interpretation of “normal” or “otherwise normal” ECG” had a NPV of 100%. No patients with these ECGs had a final diagnosis of STEMI or ACS.

This study suggests that Marquette 12SL machine-interpreted “normal” or “otherwise normal” may safely rule out STEMI or other acute signs of ACS needing immediate cardiac catheterization. This finding adds to a growing body of evidence from smaller studies that immediate emergency physician interpretation of triage ECGs with this computerized interpretation may be safely deferred until the time of patient evaluation.^7^ ^–^ ^10^ While other research has focused on a computerized interpretation of “normal” ECG this study is one of the first investigations of the reliability of a computerized interpretation of “otherwise normal” ECG. Previous research has demonstrated that immediate emergency physician interpretation of triage ECGs to screen for STEMI is time-consuming for physicians and support staff.^13^ By using this time to more directly perform patient-centered care departments could alleviate interruptions in workflow and improve patient safety.

## LIMITATIONS

While this study includes one of the largest cohorts yet of similar studies, it is limited to a single academic institution using a single type of ECG machine (Marquette 12SL). Thus, the findings may not be generalizable to other institutions and ECG machine interpretation algorithms.^4^ We chose to use a board-certified cardiologist’s final interpretation as the gold standard of ECG interpretation because this is the commonly accepted standard. Originally, we designed the study so that ECGs that had a computerized interpretation of “normal” or “otherwise normal” but a cardiologist interpretation of STEMI or a final hospital diagnosis of ACS would be reviewed by blinded, board-certified emergency physicians; however, as there were none in this large sample, this step was unnecessary. Moreover, we know from chart review, disposition, and diagnosis that none of these ECGs had an interpretation of STEMI by the emergency physician who evaluated the patient in real time.

Given that we focused on the NPV of normal computer-interpreted ECGs, we did not collect data on abnormal computer-read records. Thus, we are unable to report estimates of all screening characteristics (eg, sensitivity, specificity, and positive predictive value). The NPV of computer-read ECGs is the only characteristic reported in the study. We did not conduct follow-up after the index hospital visit and, therefore, cannot comment on 30-day major adverse cardiac events in this population. The safety of this approach is dependent on the lower bound of the CI of the sensitivity for STEMI. While this study addresses the outcome of STEMI addressed by the AHA’s guideline, it does not directly address other outcomes of interest to an emergency physician such as acute coronary occlusion MI (OMI) which may benefit from timely reperfusion therapy and is not meant to encourage physicians to forgo physician ECG interpretation even at the time of physician interpretation. Moreover, there is a growing body of literature supporting a paradigm shift from evaluating ECGs for STEMI vs no STEMI as an indicator of OMI that may benefit emergent reperfusion to evaluating ECGs for signs of acute total OMI (inclusive of STEMI negative OMI) vs non-OMI.^14^ ^,^ ^15^

## CONCLUSION

In our study population, Marquette 12SL ECG machine interpretations of “normal” or “otherwise normal” ECG excluded STEMI. Electrocardiograms with these computerized interpretations could safely wait for physician interpretation until the time of patient evaluation without delaying an acute STEMI diagnosis.
